# Creating the Healthcare Racial Justice Assessment Tool (HC-RJAT): A Novel Tool to Identify Modifiable Loci of Structural Racism in the Healthcare Setting to Guide Equity-Focused Improvements

**DOI:** 10.1007/s40615-025-02516-4

**Published:** 2025-06-24

**Authors:** Yarden S. Fraiman, Jeannette C. Myrick, Caroline Kagan, Dina Beauchamp, Peter Blades, Rachel Copertino, Elysia Larson, Cicely Fadel

**Affiliations:** 1 Department of Neonatology, Beth Israel Deaconess Medical Center, Boston, MA USA; 2 Present Address: Department of Pediatrics, Harvard Medical School, Boston, MA USA; 3 Department of Obstetrics and Gynecology, Beth Israel Deaconess Medical Center, Boston, MA USA; 4 Department of Obstetrics, Gynecology, and Reproductive Health, Harvard Medical School, Boston, MA USA

**Keywords:** Structural racism, Institutional racism, Equity, Disparity, Justice

## Abstract

Racial and ethnic health inequities are pervasive in the US healthcare system and affect individuals at all stages of life. These inequities are the result of racism and not due to biology. Racism is a multi-level system that embodies institutional, structural, interpersonal, and internalized racism. Racism in the healthcare setting creates hierarchies of power based on the social construct of race that impact opportunity, access, quality, and experiences of minoritized individuals. Most interventions to address health inequity in the healthcare setting target individual-level racism, but this has largely been ineffective in closing the equity gap. Few interventions have been developed to target structural and institutional racism. This may in part be due to a lack of healthcare-specific tools that can identify targets for intervention at the structural and institutional level. In this article, we report the development of the Healthcare Racial Justice Assessment Tool (HC-RJAT), a novel tool specifically designed to identify modifiable loci of structural and institutional racism within the healthcare setting to guide equity-focused interventions.

**Supplementary Information:**

The online version contains supplementary material available at https://doi.org/10.1007/s40615-025-02516-4.

## Introduction

Racial and ethnic health inequity is a pervasive and persistent problem in the USA and affects individuals across the entire life-course [1–3]. For the purposes of this paper, we use the term “minoritized” to refer to individuals who are non-white, similar to the more conventionally used term, “minority.” We believe “minoritized” more accurately acknowledges both the history of oppression and marginalization of non-white individuals and communities than the term “minority” while also acknowledging the active, ongoing process of discrimination through the interconnected systems of structural, institutional, interpersonal, and internalized racism.

Minoritized birthing people, in particular Black non-Hispanic birthing people, are over three times as likely to die from pregnancy-related complications [4]. Infants born to Black and Hispanic/Latinx individuals have an increased risk of having a premature or low birth weight infant, and once born, these infants are at an increased risk of adverse outcomes, including death [5–12]. Minoritized children consistently receive lower quality care and have worse health across a variety of outcomes [13, 14]. And, later in adulthood, inequities continue to persist across a vast array of health and health-related outcomes [2].

Notably, the disparate health outcomes across racial and ethnic groups are not the result of biology or genetics. Instead, health inequities are the result of racism at the micro- and macro-level [13, 15–19]. At the micro-level, internalized and interpersonal racism affect how individuals see themselves and act in the world while macro-level racism—structural and institutional racism—refers to the systems of society that uphold racialized power structures [15]. Structural racism refers to the “totality of ways in which societies foster racial discrimination through mutually reinforcing systems of housing, employment[…and] health care[.]” [17]. Institutional racism is the racism that is embedded in the laws and policies of a specific context, institution, or society [16].

Within the healthcare system, all forms of racism conspire to maintain and magnify health inequity. Racism is widespread, with over 30% of Black Americans having experienced racial discrimination at a doctor’s office and 22% of Black Americans having avoided seeking medical attention because of the fear of discrimination [20]. At the micro-level, internalized racism may influence a patient’s likelihood to speak up regarding their concerns or impact their trust in their medical care team. Interpersonal racism may affect interactions between minoritized patients and their healthcare team and is the locus of bias, both implicit and explicit, that influences care decisions. Structural racism within the healthcare system refers to the ways in which healthcare systems foster racial discrimination through reinforcing systems that include the physical environment, resources available for minoritized patients and their families, and workforce diversity, which may impact a patient’s sense of belonging and has been shown to increase uptake of medical service and compliance with medical recommendations [21, 22]. Finally, institutional healthcare racism refers to policies within the healthcare system that disproportionately affect minoritized patients and their families. For example, limitations on visiting hours, while seemingly impartial to race, disproportionately impact shift workers who themselves are predominantly minoritized. In addition, societal drivers outside the healthcare system, such as neighborhood residential segregation, further contribute to racial and ethnic health inequities [23–25].

There are also significant within-hospital inequities, even at high-performing hospitals and medical centers [26–31]. The drivers of health inequity within healthcare systems—structural and institutional racism—might be effective targets for closing the equity gap.

However, despite increased awareness of racism as the primary driver of health inequity, most health equity efforts and research continue to focus on micro-level racism, particularly interpersonal racism. This focus is evident in common approaches to address inequity which include upstander and anti-bias training and social determinants of health/social needs screening [17, 32–36]. Micro-level or individual-level interventions are important but may lead to a depletion of resources to address macro-level racism and may obscure the upstream drivers of individual-level needs [37, 38]. Furthermore, focusing on individuals may lead health systems to believe the work is complete when improvements at the structural and institutional level are necessary [38]. In other words, individual-level attention gives rise to individual-level solutions that may ultimately prove ineffective, as individual-level interventions alone have yet to close the equity gap [39, 40].

Systems thus need to create interventions specifically targeting the macro-levels of racism, structural and institutional racism, in the healthcare setting. To achieve this, we sought a systematic approach to identify key areas for intervention and to measure progress over time. In the absence of a known tool specifically designed to identify potential areas for healthcare system improvement at the structural and institutional level, we developed a novel tool to fulfill this need. In this paper, we report on the process of creating a tool designed specifically for healthcare settings to identify modifiable loci of racial and institutional racism. We use GUIDED, a guideline for reporting intervention development studies, to report the process of development and our results [41].

## Developing the Healthcare Racial Justice Assessment Tool (HC-RJAT)

### Context

The development of the HC-RJAT was based in the Department of Neonatology and the Neonatal Intensive Care Unit (NICU) at a large, level 3, academic tertiary care hospital in Boston, MA. The NICU includes a large and diverse staff comprised of attending physicians, medical trainees, advanced practice practitioners, clinical nurses, respiratory therapists, nutritionists, pharmacists, occupational therapists, patient care associates, unit coordinators, and environmental staff. The Department of Neonatology equity committee led the development of the HC-RJAT.

### Creating a Team

Within our pre-existing departmental equity committee, we formed a working group specifically focused on developing a tool to identify areas of potential equity-focused improvement at the structural and institutional level. We recognized the importance of diverse representation in increasing productivity and efficiency and supporting our anti-racism initiative. The multidisciplinary committee was formed in March 2022 and included physicians, nurses, nurse practitioners, a family partner, a unit coordinator, and patient care associate. The five-member team also included the Department of Neonatology Associate Director for Health Equity. Individuals in the working group represented white, Black, and Hispanic/Latinx racial and ethnic identities; straight and gay sexual orientation identities; and male, female, and non-binary gender identities.

### Literature Search

We began by performing a literature search to identify any existing tools to identify areas for improvement that target structural and institutional racism. Our search focused on identifying tools that (1) focused on structural or institutional racism and (2) had measurable and (3) modifiable outcomes. We identified six unique tools, including the *Racial Justice Assessment Tool* [42], *Building Organizational Capacity for Social Justice: Framework, Approach & Tools* [43], *Ready for Equity in Workforce Development: Racial Equity Readiness Assessment Tool* [44], and *Tool for Organizational Self Assessment Related to Racial Equity* [45], that met our criteria. However, none was specifically tailored to the healthcare setting [42–47].

## The Healthcare Racial Justice Assessment Tool (HC-RJAT)

We used the tools identified in the literature search to inform the development of a tool that is tailored for use in a healthcare setting. We used group discussion and iterative revisions until consensus was reached to create the Healthcare Racial Justice Assessment Tool (HC-RJAT).

The HC-RJAT is a 60-item inventory, across seven primary domains: (1) Commitment, Leadership & Governance; (2) Organization Culture; (3) Racial Equity Policies and Implementation Practices; (4) Family Facing Objectives; (5) Workforce Composition & Quality; (6) Community Collaboration; and (7) Data, Metrics, & Continuous Quality Improvement (Table 1). The HC-RJAT items each assess a potential area of healthcare structural and institutional racism. Items are scored on a three-point scale, “Red” (Not Yet), “Yellow” (In Progress), or “Green” (Fully In Place) (Supplementary Table 1). Additionally, each question can be answered by the user as “Gray” (I don’t know) to ensure accuracy and to help identify the degree of knowledge sharing and awareness. Each item maps onto one of four intervention categories: (1) Sharing & Communicating Goals; (2) Resources To Do The Work (with three sub-domains (i) Time, (ii) Funding & Compensation, and (iii) People & Training); (3) Community Involvement; and (4) Family & Patient Focused (Supplementary Table 2).

**Table 1:** Domains and sample items from the Healthcare Racial Justice Assessment Tool

Domain: Commitment, Leadership & Governance
Does the clinical space have a mission statement that incorporates racial equity?
Does the clinical space have specific staff to address racial and ethnic inequities?
Does the clinical space have compensation for staff of color to acknowledge any additional work they may absorb to better serve families of color?
Domain: Organizational Culture
Does the clinical space have visible signs of your organization’s commitment to racial equity in your primary physical location, e.g., signage that states your commitment and has representation of diverse communities?
Does the clinical space materials assessed for racial bias to ensure reflection of your community’s diversity?
Domain: Racial Equity Policies & Implementation Practices
Does the clinical space have a written racial equity plan with clear actions, timelines, people response for each action, indicators of progress and processes for monitoring and evaluation?
Does the clinical space have benchmarks around racial justice incorporated into the annual evaluation for all employees?
Does the clinical space have a definition of family that supports all family formations, including those beyond “traditional” or “nuclear” families?
Domain: Family Facing
Does the clinical space display art that is representative of families of different races?
Does the clinical space have important signage posted in the most prominent languages of the populations served?
Does the clinical space provide language interpreter/translator services for people who speak languages other than English?
Domain: Workforce Composition & Quality
Does the clinical space have hiring practices to address racial and ethnic inequities, prioritizing the hiring of employees who represent communities of color, immigrants, and refugees?
Does the clinical space have an internal structure or position dedicated to promoting workforce diversity?
Does the clinical space have racial equity and cultural competency training that is mandatory?
Does the clinical space have formal partnerships with organizations of color?
Does the clinical space have a method in place to assess the overall satisfaction of communities of color with your organization?
Domain: Data, Metrics & Continuous Quality Improvement
Does the clinical space collect data on family satisfaction with regard to racial equity?
Does the clinical space stratify clinical outcomes by race and ethnicity?
Does the clinical space allow patients and families to self-identify their race and ethnicity?

**Fig. 1:**
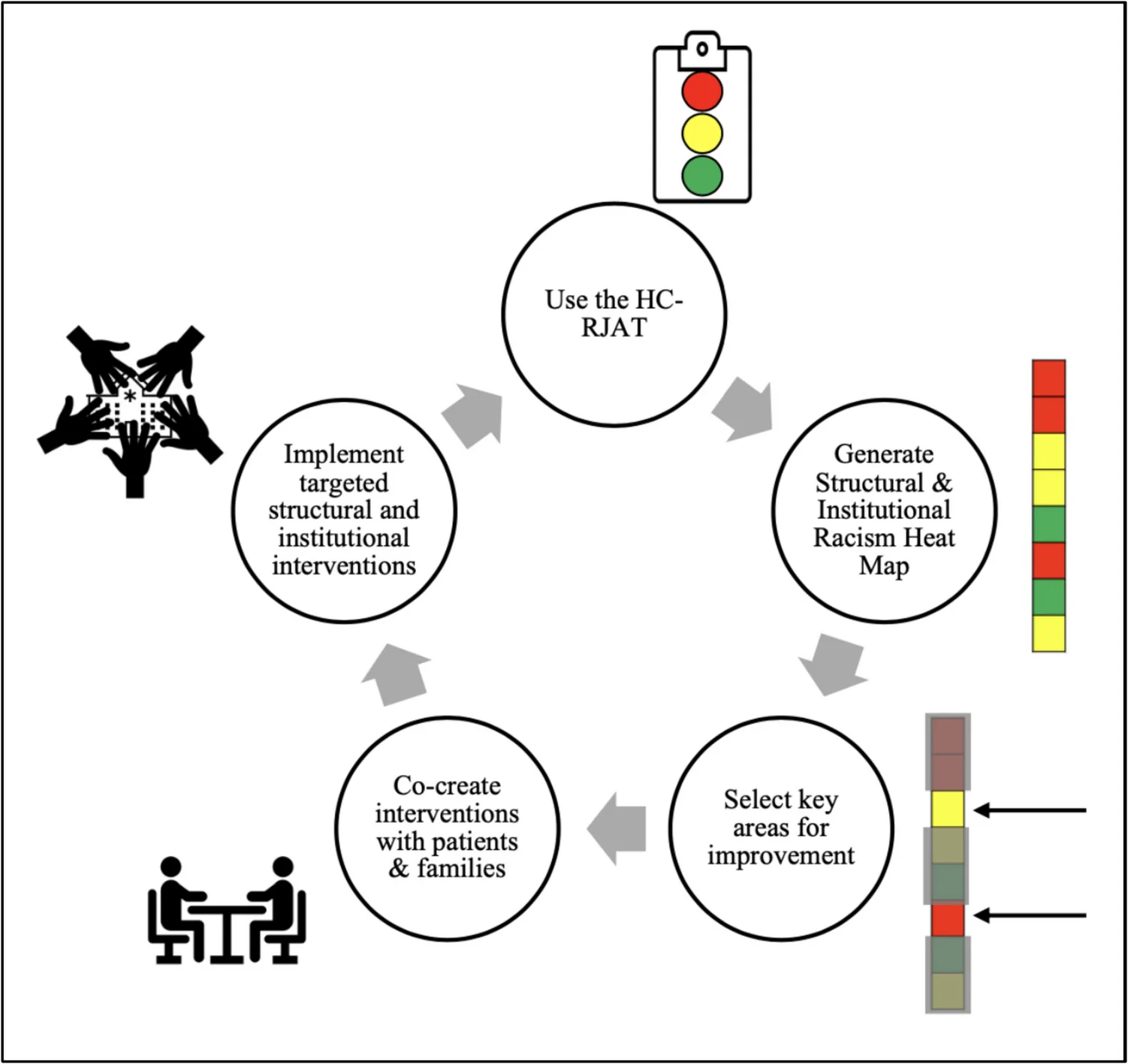
The HC-RJATas an equity-focused quality improvement tool

The HC-RJAT was designed to be completed by a group of clinical leaders and knowledge holders of the clinical spaces but can be completed by a single individual, a group of individuals, or by an organization. The responses generate a “HC-RJAT Structural and Institutional Racism Heat Map,” which produces a visual representation of the degree and extent of structural and institutional racism within the healthcare setting and across domains. The heatmap can then be used to target under-performing areas with equity-focused structural and institutional interventions. The HC-RJAT is designed to be used iteratively, using Quality Improvement (QI) methodology, to track the impact of intervention on structural and institutional racism over time and to help identify new areas for improvement (Fig. 1). For example, the clinical leaders walk through the healthcare space with the HC-RJAT and in reviewing the “HC-RJAT Structural and Institutional Racism Heat Map,” note that “Family Facing Objectives” is the domain that is the predominantly red and decide to prioritize it in their intervention. They explore each item and note that many of the questions of the physical environment (structural racism) need improvement. They partner with minoritized patient families to make changes to the environment to make it more inclusive including changing the art to show different family structures (Supplemental Table 1; HC-RJAT item #33) and varied racial and ethnic representation (Supplemental Table 1; HC-RJAT item #34). They also translate important signage to the most prominent languages of the population served (Supplemental Table 1; HC-RJAT item #35). Additionally, they address policies (institutional racism) by establishing a standard operating procedure for reviewing policies and their impact on minoritized families (Supplemental Table 1; HC-RJAT item #39) and create a system for families to report experiences of racism while in the healthcare setting (Supplemental Table 1; HC-RJAT item #43). The team thus created structural and institutional changes, guided by the HC-RJAT results and minoritized patients and families, to promote health justice. The team then uses the HC-RJAT again and compares their “HC-RJAT Structural and Institutional Racism Heat Map” to their original and note improvement overall, and specifically in the “Family Facing Objectives Domain” as a result of their interventions. In this way, using the HC-RJAT repetitively over time, users can see the impact of their interventions and seek to ascertain ongoing areas for improvement (Table 2.). From the initial meetings until finalization and use of the HC-RJAT, the process of development spanned 10 months.

**Table 2.**
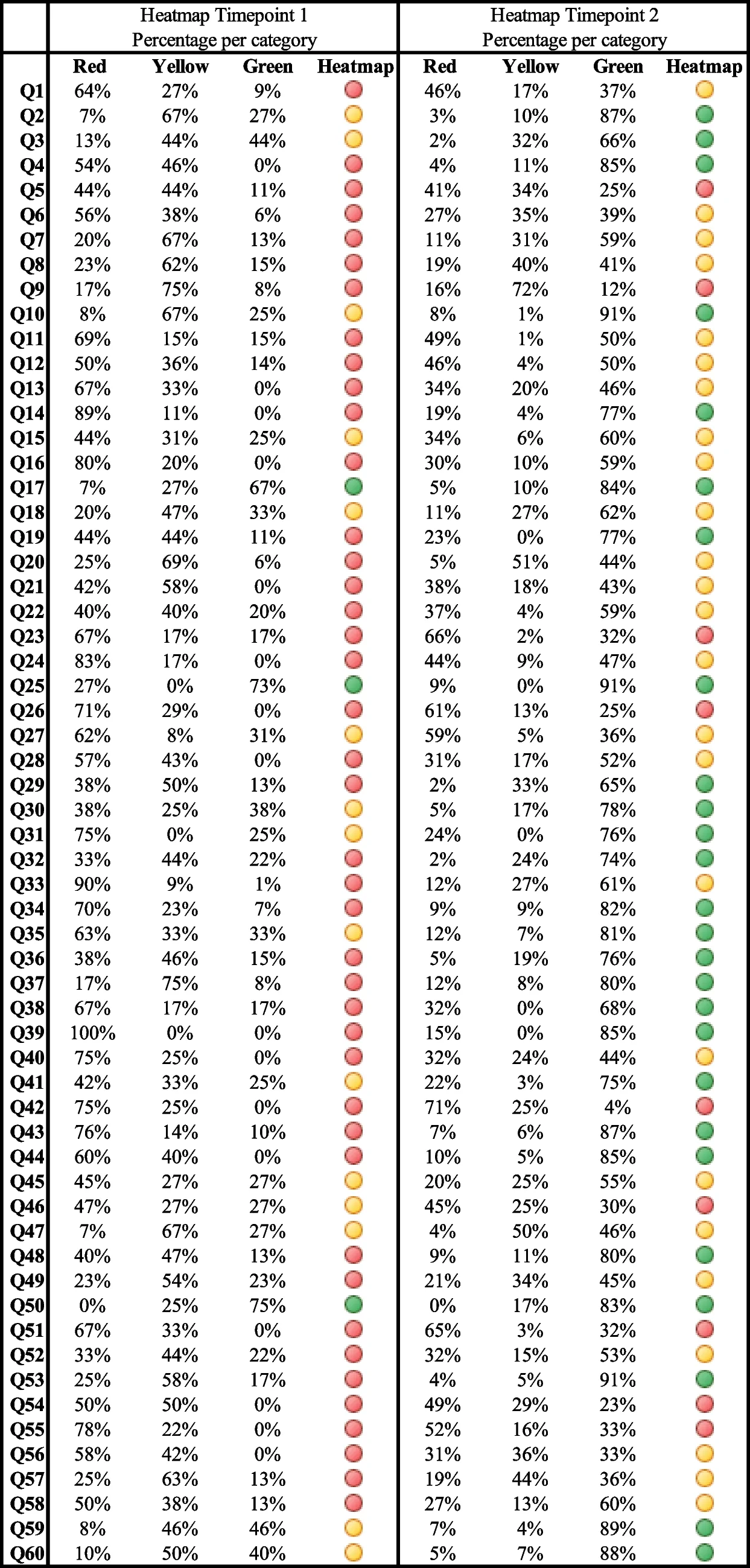
Sample HC-RJAT "Structural and Institutional Racism" Heat Map with Changes Over Time

## Understanding the Development and Use of the HC-RJAT

To document the process of developing the HC-RJAT and to elucidate the core tenets of the resulting tool, a research team (JCM) member who was not a part of the tool development process facilitated qualitative interviews with the working group. These interviews were conducted as either a 1-h focus group discussion or a one-on-one interview after the development and implementation of the HC-RJAT via Zoom. There was one focus group discussion which included all members except for YSF who was interviewed separately so as to ensure psychological safety among the team members, given his role as the team leader. The interview guide was developed prior to the interview using the updated Consolidated Framework for Implementation Research [48] (see Supplement 3). Both the interview and the discussion were recorded and transcribed. Two independent coders (JCM and CK) used a rapid analysis framework to analyze and code the transcripts [49]. Focus group and interview participants were offered $30 for participation. After completing the qualitative coding individually, the coders reached a consensus on the key themes that emerged from the developers’ interviews. Themes were discussed and refined in collaboration with YSF and EL to define fidelity of the HC-RJAT and to identify recommendations for future adaptations [50]. JCM and CK are non-physician researchers who did not participate in the development of the HC-RJAT. YSF is a physician researcher and led the team that developed the HC-RJAT. EL is a non-physician implementation scientist who did not participate in the development of the HC-RJAT. The team included cis-gendered men and women, white, Black, and Hispanic/Latinx individuals, and individuals who identify as straight and gay. This study was deemed exempt by the Institutional Review Board at Beth Israel Deaconess Medical Center.

### Reflections on the Development Process for the HC-RJAT

The HC-RJAT working group described the tool’s development process, first expressing appreciation for the team’s diverse racial and ethnic representation spanning various roles in the NICU. The team highlighted the sense of camaraderie experienced during tool development and the collaborative nature of the process and acknowledged the daunting nature of creating such a novel tool. One developer emphasized the excitement around the HC-RJAT’s development process, stating “It’s also one of those things that was […] created during this moment of a lot of interest and excitement around what could we do to close the equity gap. And what does structural institutional racism look like. And so, it felt like this was really exciting, right, to be kind of at the forefront of creating something.” The team noted their success in sourcing and analyzing various tools to fine tune the questions to feature in the tool, as well as obtaining and compiling data. The only area for improvement explicitly noted in the tool’s development process was the desire for protected and respected space to dedicate time and resources to this work, in addition to the team’s other professional and personal commitments.

### Understanding the Core Components of the Healthcare Racial Justice Assessment Tool

Preserving the core elements of the HC-RJAT is crucial to ensure that future adaptations maintain fidelity [50]. There were three central components that emerged from discussions with the development team. First is the tool’s singular focus on structural and institutional racism. One developer expressed the importance of this focus, stating “I feel like we had a very clear vision of what we were doing when we created it[…]looking specifically at structural institutional racism [was] always there at the beginning, like from the very beginning.” Second is the instructive and action-oriented nature of the tool’s questions. For example, one question prompts, “Does the [clinical space] have a mission statement that incorporates racial equity?” guiding users to create such a statement if it does not already exist in the clinical setting. Another focuses on structural interventions, asking, “Does the [clinical space] display art that is representative of the families of different races?” and “Does the [clinical space] have important signage posted in the most prominent languages of the populations served?” prompting users to ensure that the physical space of the clinical space is accessible and welcoming to the populations served. Third is the ability to use the HC-RJAT iteratively. One developer stated, “I appreciated […] that it’s iterative, so that it’s going to be something that will continue to cycle.”

### Considerations for Implementation and Adaptation of the HC-RJAT

When considering future implementation of the HC-RJAT, the development team recommended including key leaders and knowledge holders in adaptation decisions. The team also reflected on the importance of recognizing and adapting to competing priorities and changes that might impede prompt and seamless implementation in clinical settings. However, one developer underscored the critical nature of prioritizing the tool, stating “clinical care takes precedence. But, I think that’s a real gap in people’s understanding of how much this work could actually affect clinical outcomes.” The HC-RJAT developers concluded that fidelity would be achieved if adaptations of the HC-RJAT maintained its iterative and instructive nature and its focus on institutional and structural racism.

## Discussion

In this paper, we describe the development of a novel healthcare-specific tool to identify modifiable loci of structural and institutional racism within healthcare settings as a guide for macro-level equity-focused interventions. This is important as macro-level interventions to address structural and institutional racism may be the key to closing the health equity gap. However, modifiable loci of structural and institutional racism can be difficult to identify, and healthcare systems are a unique context for which existing tools may not be effective.

We report the development of the Healthcare Racial Justice Assessment Tool (HC-RJAT), a novel tool to identify modifiable loci of structural and institutional racism within healthcare settings. Developed by a multidisciplinary and diverse team, the HC-RJAT integrates the unique perspectives of multiple healthcare knowledge holders.

The HC-RJAT was designed in the setting of a paucity of healthcare-specific tools to measure structural and institutional racism. Consider that “what gets measured is what gets done,” the HC-RJAT is the first step in evaluating a clinical space to identify the modifiable loci of healthcare-based structural and institutional racism. The tool generates a structural and institutional racism heatmap that can be used to identify the items and domain that are most in need of interventions. As each item is action-oriented and instructive in nature, developing interventions to create structural and institutional health justice is directed, often overcoming institutional barriers related to lack of expertise and personnel. Since the HC-RJAT can be used iteratively, changes in the healthcare environment can be tracked over time, and the effectiveness of interventions can be monitored (Table 2.). We recommend establishing a regular cadence of using the HC-RJAT, examining the heatmap, and designing and implementing interventions in a cycle of continuous improvement. For example, the NICU conducts the HC-RJAT annually, reviewing the heatmap and prioritizing interventions to consistently monitor progress and make iterative improvements within the unit. We encourage institutions to establish a cadence that is feasible and sustainable for them. Additionally, when developing interventions, users can adapt existing effective interventions and approaches [51].

One limitation of the HC-RJAT is the lack of generalizability to contexts outside the healthcare setting. However, given the vast number of tools available to examine macro-level racism in other contexts, and that informed the HC-RJAT development, we believe that this limitation is overcome by existing tools and resources. Additionally, not every item on the HC-RJAT may be generalizable to each healthcare context and was developed by members of a single department. We expect that the HC-RJAT will be adapted, and we encourage users to employ Implementation Science methods, which provide a rigorous approach to understanding why certain interventions work and are efficient in unique contexts. Moreover, adaptation can lead to “improved engagement, acceptability, and clinical outcomes, particularly when working with minority populations” [52]. Structural and institutional racism persists throughout the US healthcare system in varied clinical settings, and context-specific and fidelity-consistent adaptations of this tool are welcome and necessary to utilize the HC-RJAT to address these inequities. When adapting the tool, we recommend users to employ a systematic approach, such as the expanded framework for reporting adaptations and modifications to evidence-based interventions (FRAME) [52]. Notably, the HC-RJAT was not designed to address structural and institutional racism outside of the healthcare system which is also an important driver of health inequity within the healthcare system. However, the tool is designed to uncover, address, and track macro-level racism within healthcare settings and is the first tool of its kind to do this. We acknowledge that the focus of the HC-RJAT is healthcare-based macro-level racism and does not address drivers of health inequity that minoritized patients and communities encounter outside the healthcare system.

Furthermore, we acknowledge that the HC-RJAT was developed with limited patient family input, an essential factor in all healthcare-based interventions, especially those designed to promote health equity and justice. While structural and institutional racism have immediate impacts on patient experiences within the healthcare system, patients and families are often not aware of existing policies and systems within the healthcare system. As such, the HC-RJAT was not designed for the patient or family to be the end user. However, we encourage future users of the HC-RJAT to more fully involve patients and families when prioritizing areas for interventions informed by the HC-RJAT and in designing equity-focused patient-facing interventions [53–55]. Future research can focus not only on the impact of the HC-RJAT on healthcare-based structural and institutional racism, but also on outcome measures including patient- and family-reported experience and outcome measures and quantitative health outcomes.

A notable strength of this work is the rigorous and reflective process we employed to examine the development of this novel tool and to define fidelity for future adaptations. While the members of the working group are all co-authors on this work, additional researchers and implementation scientists external to the working group conducted focus group discussions and interviews and critically analyzed the results. Additional care was taken in the qualitative approach, and YSF, who has a leadership role in the Department of Neonatology, was interviewed separately to best ensure that all members of the working group had the psychological safety and security to reflect on their experiences. The multidisciplinary collaboration across roles and identities in the development of the HC-RJAT is a particular strength which acknowledges the hierarchical nature of medicine and of society.

Potential future directions include the validation of the HC-RJAT in varied clinical settings, the exploration of patient-reported experience and outcome measures as a result of HC-RJAT-guided interventions, and studies to measure the impact of addressing healthcare-based structural and institutional racism on health outcomes and health inequity.

## Conclusion

The Healthcare Racial Justice Assessment Tool is a novel tool designed by a multidisciplinary and diverse working group to identify modifiable loci of structural and institutional racism in the healthcare setting. This tool can be used to inform equity-focused healthcare interventions specifically targeting macro-level racism, as opposed to individual-level racism. The HC-RJAT directs healthcare systems to structural and institutional loci of change, which may be the key to closing the pervasive racial and ethnic health equity gap.

## Supplementary Information

Below is the link to the electronic supplementary material.

ESM 1 (PDF 67.1 KB)

ESM 2 (PDF 61.1 KB)

ESM 3 (DOCX 20.3 KB)

## Data Availability

Not applicable.
