# Supplementary material for: Creating the Healthcare Racial Justice Assessment Tool (HC-RJAT): A Novel Tool to Identify Modifiable Loci of Structural Racism in the Healthcare Setting to Guide Equity-Focused Improvements
Source: J Racial Ethn Health Disparities. 2025 Jun 24;13(5):3556–65. doi: 10.1007/s40615-025-02516-4 (PMC13546341; doi:10.1007/s40615-025-02516-4)
Supplement: Supplementary file 1 [file 40615_2025_2516_MOESM1_ESM.pdf]

Supplementary Table 1. The Healthcare Racial Justice Assessment Tool (HC-RJAT)

| Domain | | Does the healthcare setting have… | Red<br>Not Yet | Yellow<br>In Progress | Green<br>Fully In Place | Gray<br>I'm Not Sure |
|---|---|---|---|---|---|---|
| Commitment, Leadership & Governance | 1 | A mission statement that incorporates racial equity? | | | | |
| | 2 | An internal structure whose goal is to address issues of racial equity, for example an equity committee? | | | | |
| | 3 | Specific staff to address racial and ethnic inequities? | | | | |
| | 4 | Categorized funds used to support programs/policies needed by particular disadvantaged racial/ethnic communities? | | | | |
| | 5 | Leadership that consistently applies a racial equity lens? | | | | |
| | 6 | A view that advocacy for racial equity is part of the organization's work? | | | | |
| | 7 | A planned and periodic gathering of facts and governing body discussion with community participation to review racial equity? | | | | |
| | 8 | A deliberate plan to develop and promote the leadership of staff of color? | | | | |
| | 9 | Regular trainings and discussions for all staff levels about removing barriers to opportunity and reducing racial disparities? | | | | |
| | 10 | Processes in place to ensure all staff can implement cultural competence/responsiveness and trauma-informed approaches? | | | | |
| | 11 | Compensation for staff of color to acknowledge any additional work they may absorb to better serve families of color? | | | | |
| | 12 | A balanced view that works to involve, but not overburden, our staff of color in equity work? | | | | |
| Oragnizational Culture | 13 | A process to consistently consider basic needs like childcare, interpretation, food, proximity to transit lines, or time of day when planning activities and events for staff? | | | | |
| | 14 | A process to consistently consider basic needs like childcare, interpretation, food, proximity to transit lines, or time of day when planning activities and events for patients? | | | | |
| | 15 | A process to consistently communicate its Racial Justice values and work to staff? | | | | |
| | 16 | A process to consistently communicate its Racial Justice values and work to patients/families? | | | | |
| | 17 | A process to consistently communicate its Racial Justice values and work to donors? | | | | |
| | 18 | Visible signs of your organization's commitment to racial equity in your primary physical location, e.g. signage that states your commitment and has representation of diverse communities? | | | | |
| | 19 | Materials assessed for racial bias to ensure reflection of your community's diversity? | | | | |
| | 20 | A mechanism to encourage or support real time difficult conversations about race in a safe, confidential, private space? | | | | |
| | 21 | Provision of space, time, resources, and structure to discuss and respond to issues of Racial Justice within your organization? | | | | |
| | 22 | A shared language and understanding around the concept of equity? | | | | |
| Racial Equity Policies and Implementation Practices | 23 | A written racial equity plan with clear actions, timelines, people responsible for each action, indicators of progress and processes for monitoring and evaluation? | | | | |
| | 24 | Participation of representatives from communities of color when developing policies? | | | | |
| | 25 | Metrics, benchmarks, and indicators for measuring the organization's success? | | | | |
| | 26 | Benchmarks around racial justice incorporated into the annual evaluation for all employees? | | | | |
| | 27 | Anti-discrimination policies that explicitly prohibit harassment of persons of color who are members of the organization? | | | | |
| | 28 | A definition of family that supports all family formations, including those beyond "traditional" or "nuclear" families? | | | | |
| | 29 | Periodic assessment of the disproportionate impact of organizational policies on staff of color? | | | | |
| | 30 | Periodic assessment of the disproportionate impact of organizational policies on patients and families of color? | | | | |
| | 31 | A process for staff to provide feedback to the hospital about its larger policies in a way that is equity-minded? | | | | |
| | 32 | Regular communication with other DEI groups throughout the larger organization? | | | | |
| Family Facing Objectives | 33 | Have a physical environment that feels inclusive to all of our families? | | | | |
| | 34 | Display art that is representative of different family structures? | | | | |
| | 35 | Display art that is representative of families of different races? | | | | |
| | 36 | Have important signage posted in the most prominent languages of the populations we serve? | | | | |
| | 37 | Collect racial, ethnic and linguistic data on families using a written policy or formal practice? | | | | |
| | 38 | Provide language interpreter/translator services for people who speak languages other than English? | | | | |
| | 39 | Have a process to evaluate whether current policies disadvantage families of color? | | | | |
| | 40 | Prioritize support and referral outlets that specifically reach patients, families and communities of color or those with limited English proficiency? | | | | |
| | 41 | Address structural barriers that might prevent families of color or those with limited English proficiency from accessing your services? | | | | |
| | 42 | Have infrastructure that allows you to truly measure the positive or negative impact you are having on the lives of the families of color you serve? | | | | |
| | 43 | Have a process to support families who may be experiencing racism while accessing your services? | | | | |
| Workforce Composition & Quality | 44 | Collect the racial, ethnic and linguistic makeup of your workforce using a written policy or formal practice? | | | | |
| | 45 | Have data on the racial, ethnic and linguistic makeup of your leadership team? | | | | |
| | 46 | Have hiring practices to address racial and ethnic inequities, prioritizing the hiring of employees who represent communities of color, immigrant and refugees? | | | | |
| | 47 | Have written procedures to increase the recruitment, retention and promotion of people of color? | | | | |
| | 48 | Have an internal structure or position dedicated to promoting workforce diversity? | | | | |
| | 49 | Incorporate racial justice knowledge into performance objectives and evaluations for staff? | | | | |
| | 50 | Have effective formal complaint procedures for staff regarding race-related complaints? | | | | |
| | 51 | Have racial equity and cultural competency training that is mandatory? | | | | |
| Community Collaboration | 52 | Have formal partnerships with organizations of color? | | | | |
| | 53 | Allocate resources for engagement and outreach in communities of color? | | | | |
| | 54 | Meet regularly with leaders from communities of color specifically to discuss racial equity within your organization? | | | | |
| | 55 | Have a method in place to assess the overall satisfaction of communities of color with your organization? | | | | |
| Data, Metrics, & Continuous Quality Improvement | 56 | Collect data on family satisfaction with regard to racial equity? | | | | |
| | 57 | Collect race and ethnicity data on those referred for specific interventions, consultations, or clinics? | | | | |
| | 58 | Stratify clinical outcomes by race and ethnicity? | | | | |
| | 59 | Allow patients and families to self-identify their race and ethnicity? | | | | |
| | 60 | Allow patients and families to designate multiple races and/or ethnicities? | | | | |
