# Supplementary material for: Creating the Healthcare Racial Justice Assessment Tool (HC-RJAT): A Novel Tool to Identify Modifiable Loci of Structural Racism in the Healthcare Setting to Guide Equity-Focused Improvements
Source: J Racial Ethn Health Disparities. 2025 Jun 24;13(5):3556–65. doi: 10.1007/s40615-025-02516-4 (PMC13546341; doi:10.1007/s40615-025-02516-4)
Supplement: Supplementary file 2 [file 40615_2025_2516_MOESM2_ESM.pdf]

Supplementary Table 2. The Healthcare Racial Justice Assessment Tool (HC-RJAT) Grouped by Intervention Category

**Intervention Category**		**Does the healthcare setting have…**
Sharing/Communicating Common Goals		A mission statement that incorporates racial equity?
		Leadership that consistently applies a racial equity lens?
		A view that advocacy for racial equity is part of the organization’s work?
		A process to consistently communicate its Racial Justice values and work to staff?
		A process to consistently communicate its Racial Justice values and work to donors?
		Visible signs of your organization’s commitment to racial equity in your primary physical location, e.g. signage that states your commitment and has representation of diverse communities?
		A shared language and understanding around the concept of equity?
		Materials assessed for racial bias to ensure reflection of your community’s diversity?
		A written racial equity plan with clear actions, timelines, people responsible for each action, indicators of progress and processes for monitoring and evaluation?
		Metrics, benchmarks, and indicators for measuring the organization’s success?
		Regular communication with other DEI groups throughout the larger organization?
Resources To Do The Work	Time	A mechanism to encourage or support real time difficult conversations about race in a safe, confidential, private space?
		Provision of space, time, resources, and structure to discuss and respond to issues of Racial Justice within your organization?
	Funding & Compensation	Categorized funds used to support programs/policies needed by particular disadvantaged racial/ethnic communities?
		Compensation for staff of color to acknowledge any additional work they may absorb to better serve families of color?
		Allocate resources for engagement and outreach in communities of color?
	People & Training	An internal structure whose goal is to address issues of racial equity, for example an equity committee?
		Specific staff to address racial and ethnic inequities?
		A deliberate plan to develop and promote the leadership of staff of color?
		Regular trainings and discussions for all staff levels about removing barriers to opportunity and reducing racial disparities?
		Processes in place to ensure all staff can implement cultural competence/responsiveness and trauma-informed approaches?
		A balanced view that works to involve, but not overburden, our staff of color in equity work?
		A process to consistently consider basic needs like childcare, interpretation, food, proximity to transit lines, or time of day when planning activities and events for staff?
		Benchmarks around racial justice incorporated into the annual evaluation for all employees?
		Anti-discrimination policies that explicitly prohibit harassment of persons of color who are members of the organization?
		Periodic assessment of the disproportionate impact of organizational policies on staff of color?
		A process for staff to provide feedback to the hospital about its larger policies in a way that is equity-minded?
		Collect the racial, ethnic and linguistic makeup of your workforce using a written policy or formal practice?
		Have data on the racial, ethnic and linguistic makeup of your leadership team?
		Have hiring practices to address racial and ethnic inequities, prioritizing the hiring of employees who represent communities of color, immigrant and refugees?
		Have written procedures to increase the recruitment, retention and promotion of people of color?
		Have an internal structure or position dedicated to promoting workforce diversity?
		Incorporate racial justice knowledge into performance objectives and evaluations for staff?
		Have effective formal complaint procedures for staff regarding race-related complaints?
		Have racial equity and cultural competency training that is mandatory?
Community Involvement		A planned and periodic gathering of facts and governing body discussion with community participation to review racial equity?
		Participation of representatives from communities of color when developing policies?
		Have formal partnerships with organizations of color?
		Meet regularly with leaders from communities of color specifically to discuss racial equity within your organization?
		Have a method in place to assess the overall satisfaction of communities of color with your organization?
Family & Patient Focused		A process to consistently consider basic needs like childcare, interpretation, food, proximity to transit lines, or time of day when planning activities and events for patients?
		A process to consistently communicate its Racial Justice values and work to patients/families?
		A definition of family that supports all family formations, including those beyond “traditional” or “nuclear” families?
		Periodic assessment of the disproportionate impact of organizational policies on patients and families of color?
		Have a physical environment that feels inclusive to all of our families?
		Display art that is representative of different family structures?
		Display art that is representative of families of different races?
		Have important signage posted in the most prominent languages of the populations we serve?
		Collect racial, ethnic and linguistic data on families using a written policy or formal practice?
		Provide language interpreter/translator services for people who speak languages other than English?
		Have a process to evaluate whether current policies disadvantage families of color?
		Prioritize support and referral outlets that specifically reach patients, families and communities of color or those with limited English proficiency?
		Address structural barriers that might prevent families of color or those with limited English proficiency from accessing your services?
		Have infrastructure that allows you to truly measure the positive or negative impact you are having on the lives of the families of color you serve?
		Have a process to support families who may be experiencing racism while accessing your services?
		Collect data on family satisfaction with regard to racial equity?
		Collect race and ethnicity data on those referred for specific interventions, consultations, or clinics?
		Stratify clinical outcomes by race and ethnicity?
		Allow patients and families to self-identify their race and ethnicity?
		Allow patients and families to designate multiple races and/or ethnicities?
