# Supplementary material for: Creating the Healthcare Racial Justice Assessment Tool (HC-RJAT): A Novel Tool to Identify Modifiable Loci of Structural Racism in the Healthcare Setting to Guide Equity-Focused Improvements
Source: J Racial Ethn Health Disparities. 2025 Jun 24;13(5):3556–65. doi: 10.1007/s40615-025-02516-4 (PMC13546341; doi:10.1007/s40615-025-02516-4)
Supplement: Supplementary file 3 [file 40615_2025_2516_MOESM3_ESM.docx]

Supplement 3: Semi-structured focus group/interview guide using the updated Consolidated Framework for Implementation Research

Norming Statement: We come to this focus group with different roles and perspectives. While the practice of medicine may be hierarchical, our goal here is to collaborate as equals to reflect on how the HC-RJAT has functioned in our department to improve the experience for both the patients we serve and for each other. We will all do our best to check our formal roles at the metaphorical door, recognizing that we all bring valuable insights and have much to learn from one another. I would like to suggest the following norms for our group, called the “ABCs”:

**A:** Assume best intentions.

**B:** Brave space.

**C:** Conversation space.

 By a show of hands, do we all agree to these norms?

Focus Group Discussion/Interview Questions

1. In one sentence, please describe the core of the HC-RJAT.
2. Thank you for providing your personal definitions of the HC-RJAT. The developers describe the HC-RJAT as a tool that identifies modifiable loci of structural and institutional racism. It is designed to be used iteratively to track improvement over time. Additionally, the question items are meant to be action-oriented to direct individuals to structural and institutional level interventions. With this definition in mind, what was adapting the HC-RJAT like for you?
2. Are there alternative approaches that were not the HC-RJAT that were considered in your department?
   a. If yes, how does the HC-RJAT compare to other alternatives that may have been considered or that you know about?
   b. What advantages does the HC-RJAT have compared to these other programs?
   c. What disadvantages does the HC-RJAT have compared to these other programs?
3. Who are the key influential individuals to get on board with implementing the HC-RJAT?
   a. Who are the key people needed to greenlight (or approve) the use of this tool?
   b. Who are the key people needed to implement this tool, meaning fill out the HC-RJAT?
   c. Who are the key people to respond to the results of tool and take part in the prioritization process?
   d. Who are the key people to move the identified interventions forward?
5. What recommendations would you have for another department who wanted to adapt the HC-RJAT for their use?
6. Are there components of the HC-RJAT that should not be altered?
7. To what extent might HC-RJAT implementation take a backseat to other high-priority initiatives going on now?
   a. How important do you think it is to implement the HC-RJAT compared to other priorities?
   b. How important is it to others, such as your coworkers or leaders, to implement the HC-RJAT compared to other priorities?
8. To what extent do you think people were aware of the HC-RJAT assessment? And to what extent do you believe people were affected by its use?
   a. What are the facilitators/barriers to reaching more people in the department?
9. How well or not well do you think the HC-RJAT integrated into the OBGYN department setting?
10. Given the barriers, what do you think needs to be implemented with the HC-RJAT to ensure that it is effective?
    a. What do you think needs to be implemented to ensure the use of the tool to create change is effective?
11. How well do you think use of the HC-RJAT (by department staff and leadership) will meet the needs of the individuals served by the department?
    a. In what ways will using the HC-RJAT meet their needs?
12. What patient-level outcomes do you think could change through the use of the HC-RJAT?
13. Is there anything else you would like to share with us about the HC-RJAT or its implementation?
